# A process-based recovery indicator for anthropogenically disturbed river system

**DOI:** 10.1038/s41598-022-14542-x

**Published:** 2022-06-20

**Authors:** C. Pradhan, S. K. Padhee, Rishikesh Bharti, S. Dutta

**Affiliations:** 1 Department of Civil Engineering, Indian Institute of Technology Guwahati, Guwahati, 781039 India; 2 International Water Management Institute, Delhi, India

**Keywords:** Geomorphology, Hydrology

## Abstract

The present paper utilizes entropy theory and Google earth engine cloud computing technique to investigate system state and river recovery potential in two large sub-basins of the Mahanadi River, India. The cross-sectional intensity entropy (CIE) is computed for the post-monsoon season (October–March) along the selected reaches. Further, a normalized river recovery indicator (NRRI) is formulated to assess the temporal changes in river health. Finally, NRRI is related to a process-based variable-LFE (low flow exceedance) to comprehend the dominating system dynamics and evolutionary adjustments. The results highlight the existence of both threshold-modulated and filter-dominated systems based on CIE and NRRI variabilities. In addition, the gradual decline in CIE and subsequent stabilization of vegetated landforms can develop an ‘event-driven’ state, where floods exceeding the low-flow channel possess a direct impact on the river recovery trajectory. Finally, this study emphasizes the presence of instream vegetation as an additional degree of freedom, which further controls the hierarchy of energy dissipation and morphological continuum in the macrochannel settings.

## Introduction

Around the globe, anthropogenic stresses in terms of flow-sediment regulation, deforestation, channelization, and clearing of (instream) riparian vegetation have created both on-site and legacy effects in the fluvial systems^1–6^. The geomorphic impacts of such disturbances can vary from localised scour and incision to large scale transformation in channel patterns^7–12^. Gregory^13^ investigated different stages of human disturbance induced morphological adjustments and inspected the evolution of several pertinent concepts. One such key concept is recovery potential, which is defined as the capacity of the river to adjust to the prevailing boundary conditions^14^. River recovery is also related to the improvement of geomorphic conditions over decadal frameworks, where each reach must be analysed within its catchment context^15^. River recovery captures the past trajectories of channel adjustments and facilitates an understanding for the present state and future scenarios. The identification of evolutionary trajectories and rates of recovery are associated with historical analysis and field investigations^16^. River style framework is also developed through ergodic reasoning and analysis of the assemblage of geomorphic units defining the reach^17–19^. The synthesis of the literature suggests that stages of recovery or deterioration have been determined by the presence, absence or reconstruction of the assemblage of geomorphic units that are expected to occur for different river types^15,20–22^. However, it is challenging to identify the recovery stage of large fluvial systems with field-based evolutionary records of instream and floodplain geomorphic units.

The macrochannels are defined by pronounced ‘channel-in-channel’ physiography^23,24^ and formed by hierarchical low-flows and bankfull floods^25,26^. Many rivers in the Southeast Asia and Australia are macrochannels and show complex arrangements of fluvial features at varying flow depths^27^. The previous works on macrochannel river systems are focused on the change of flow regime^24,27–30^, alteration of channel hydraulics^31–35^, the influence of vegetation and ecological management^36–40^, sensitivity and connectivity analysis^41,42^, and understanding the channel evolution and metamorphosis^26,43–45^. Despite the well documentation of process-form-ecological relationships, understanding the direction of morphological continuum and association-feedbacks of vegetated landforms are relatively understudied in macrochannel systems^26,45^. In addition, instream vegetation emerges as dominating factor in controlling the direction of fluvial form in the macrochannels with reduced degrees of freedom. The vegetation cover is also linked with the river recovery process, and therefore, formulating a process-based river recovery indicator for macrochannel settings will provide idea about the river recovery trajectory and its underlying processes. Such understanding is crucial for river corridor management in anthropogenically disturbed river systems.

The entropy theory has been used in geomorphological studies in different scales and forms. Leopold and Langbein^46^ applied the concept of entropy in landscape evolution and developed the most probable condition based on uniform distribution of energy in fluvial systems. A similar application of entropy to river basin networks was carried out by Fiorentino et al.^47^. Their study further explored the relationship between mean elevation, potential energy and morphological characteristics of drainage basin. Gholami et al.^48^ assessed the transverse slope of bank profiles and its associated hydraulic-geometric properties using the entropy parameter. Likewise, Chembolu and Dutta^49^ developed an entropy-based planform disorder index to understand the process-form interactions of the highly braided Brahmaputra River. Other studies have utilized the entropy concept in the evolution of river delta^50,51^, flow monitoring^52^, velocity measurement^53,54^, discharge estimation^55^, and landscape stability^56^. In recent years, entropy theory has been used in river health assessment in terms of flow measurement^57^, flood control^58,59^, water supply and quality analysis^60^, ecological biomass measurement^61^, development of sustainable development goals^62^, and wastewater treatment rate quantification in urban areas^63^. Thus, entropy has emerged as an important concept in geomorphic studies. The macrochannel systems are subjected to considerable hydrological variability and erosion–deposition processes that affect the functional surfaces of geomorphic units and associated riverine health^45^. In addition, the seasonal switches of threshold-modulated processes (erosion to deposition and vice-versa) create divergent and convergent system states. The divergence or multiple endpoints of landscape evolution is problematic for river managers, and hence, convergence or single end system state is often preferred^64^. The concept of entropy emerges as an essential tool to capture these cross-sectional landscape evolution processes of macrochannel systems and provide a basis for river health trajectory assessment. In the present study, concept of entropy and integrated Google Earth Engine (GEE) cloud computation techniques are used to contribute to this challenge.

In India, peninsular rivers are an integral part of the water-food-energy nexus, supporting millions of people through agriculture, industries, and flood control^6^. However, these river basins also have a large history of receiving intense anthropogenic stresses for two centuries. For example, an extensive loss of forest cover was reported for the central-east regions of India in 1880–1960^65^. The dam-building activity was also at its peak during 1970–1990, and numbers of the small height large dams and mega-dams of national importance had increased by three to four times^6^. Such large scale anthropogenic disturbances combined with localised fluvial disturbances like sand mining, instream (riparian) vegetation loss, and channelization have instigated geomorphic and (bio)ecological adjustments along the fluvial systems^66–69^. The major impacts include variability in flow-sediment regime^70^, reach-scale channel pattern alteration^71^, coastal erosion^72^, riparian wetland area loss^73^, and salt-water intrusion^74^. However, understanding the process-form relationship in these poorly gauged-anthropogenically disturbed rivers is still in the preliminary stages and therefore, demands interdisciplinary, multifaceted approaches. The study area of this paper includes two such basins (the Ong and the Tel), where both anthropogenic and natural stressors have significantly altered the bio-morphological interactions. In addition, the integration of entropy theory with GEE cloud computation techniques will be the first attempt to develop process-based recovery indicators for the macrochannel systems. Hence, the objectives of this study are to (1) develop an entropy-based indicator to incorporate the cross-sectional disorderness, (2) assess the spatio-temporal variability in instream vegetation cover using GEE cloud computations, and (3) finally, formulate process-based recovery indicators to monitor river health and system state.

## Study area

**Figure 1:**
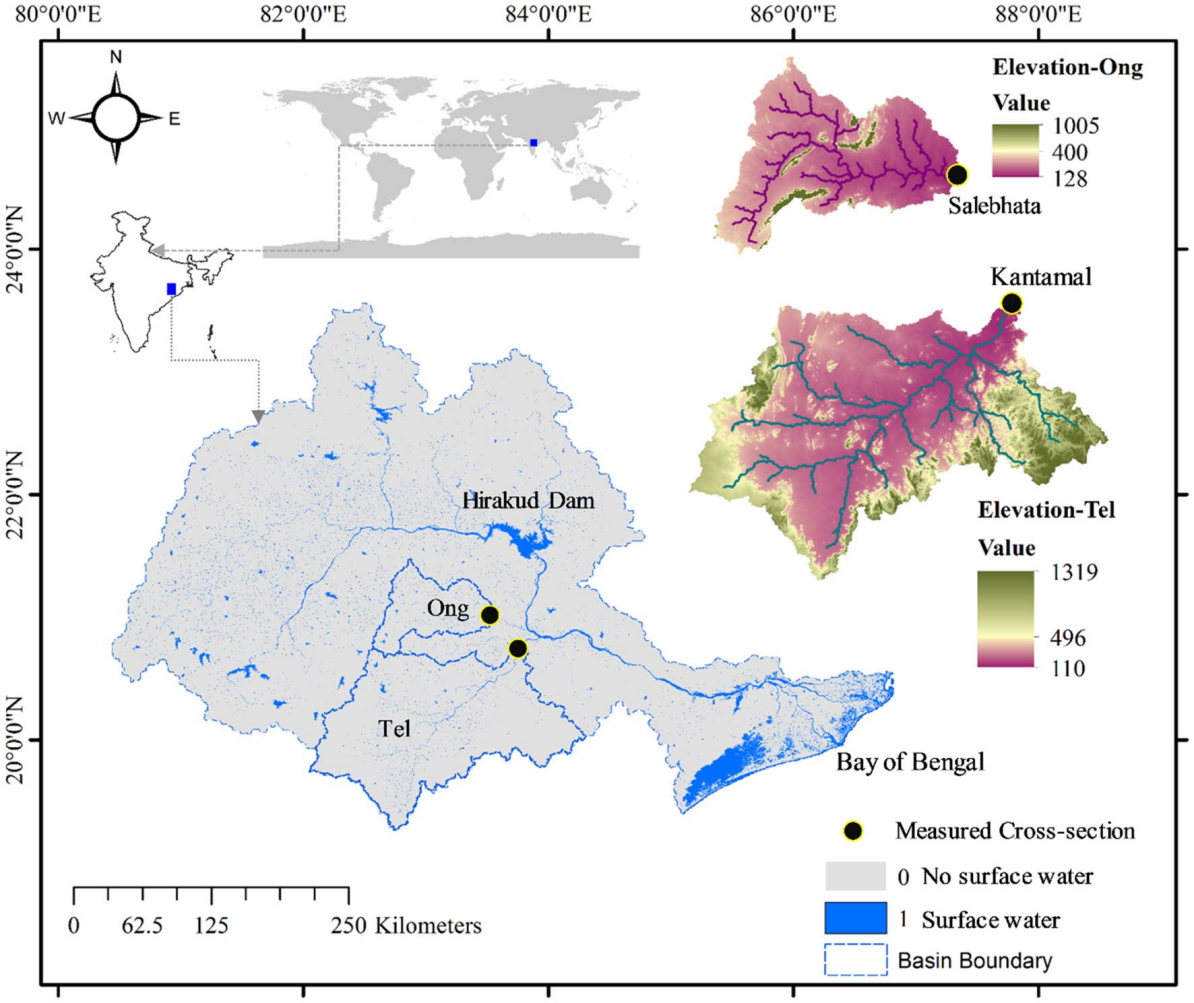
Study area showing the Ong and the Tel sub-basins of the Mahanadi River (India).

The Mahanadi River basin is the fifth largest watershed in India, covering a total geographic area of nearly 4.3% (India-WRIS). The earlier studies on the Mahanadi and its tributaries include understanding of the flow-sediment–water quality variability^75–78^, the morphological characteristics^79–81^, and the ecological entities^82–84^. The Ong and the Tel are the two largest tributaries of the Mahanadi (India), with a combined catchment area of 27, 946 km^2^ and a channel length of 184 km (Fig. 1). These rivers drain along mix red and black soils^85^. During the south-west monsoon (June to September), the river basins have an average annual rainfall of about 1463 mm. The Ong is governed by the Guchhepali dam^86^, and the Tel has a dynamic flow-sediment regime owing to the combined effects of natural and anthropogenic stresses. Two gauging stations, Salebhata and Kantamal, are present at 30 km and 40 km upstream of the Ong-Mahanadi and the Tel-Mahanadi confluences, respectively.

**Figure 2:**
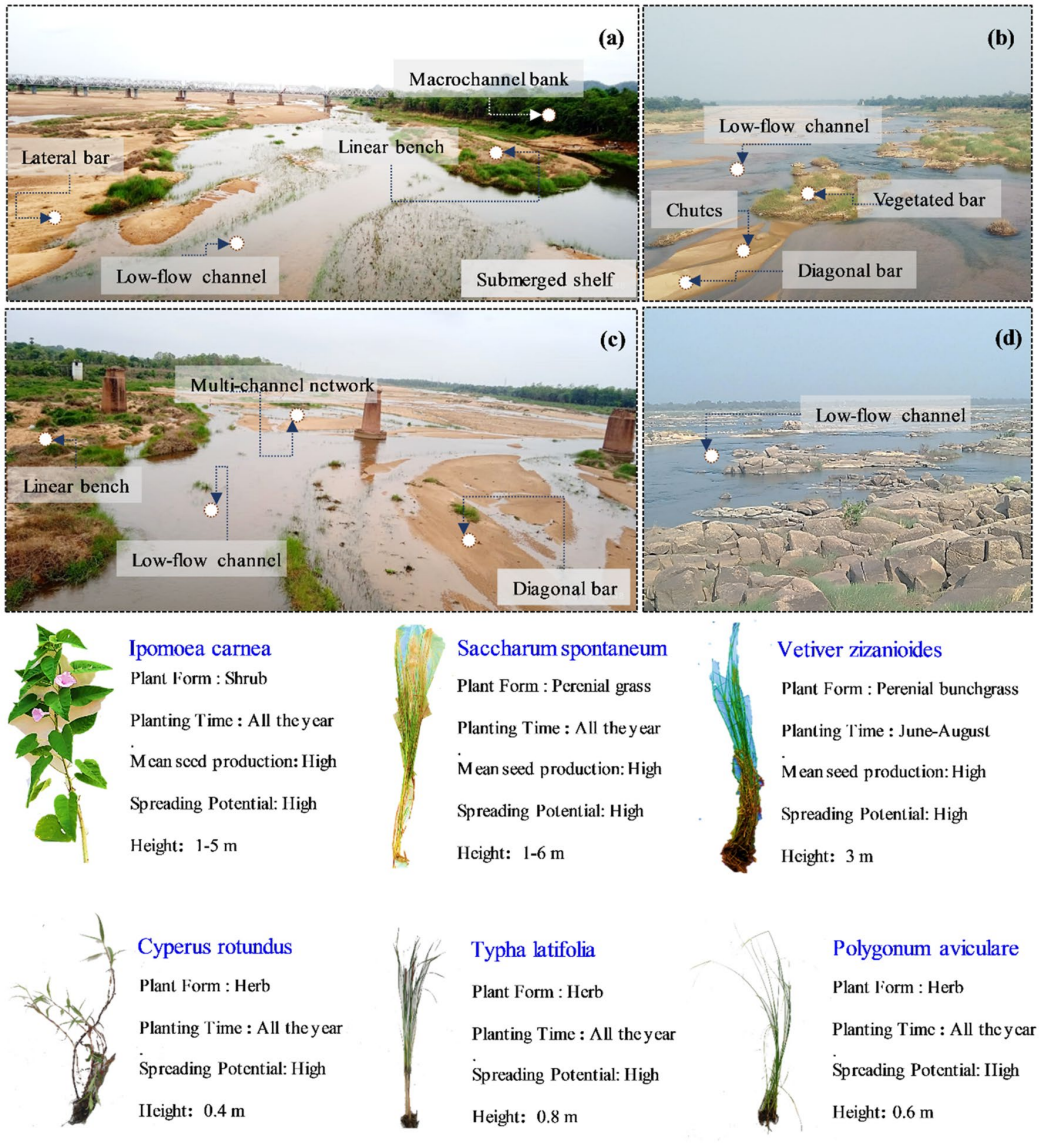
Field photographs showing the presence of instream and floodplain geomorphic units in the Ong-Tel paired catchment, (**a**,**c**) macrochannel banks and instream vegetated geomorphic units in the Ong River, and (**b**,**d**) prevalence of diagonal bars and defined low-flow channel in the Tel River. The dominant instream and riparian vegetation cover details are also shown along the two reaches.

The channel form of the Ong and the Tel is described as macrochannel, in which a smaller low flow channel is inset within a larger channel. At the measured cross-section locations, the macrochannel and inset bankfull widths are close to 0.4 and 0.1 km, respectively. The significant difference between the inset and the macrochannel dimensions offers large space for geomorphic adjustments at multiple inundation surfaces (Fig. 2). The macrochannel bank height is 8 to 10 m, which can accommodate a flow regime of high hydrological variability. Tributary inflow and bank erosion are also absent, and geomorphic units such as benches, chutes and various bar types (vegetated and unvegetated) are effectively confined between the macrochannel bank margins. The instream vegetation covers are normally shrubs, grasses, and twinners plants with soft stems, and flexible bends and have deep roots into the sand. The vegetations like *tamarix ericoides*, *coix lacryma-jobi*, *hedyotis corymbosa*, *cyperus rotundus*, *typha latifolia*, *polygonum aviculare* and *polygonum barbatum* spread profusely in the fluvial corridor. In particular, an accelerated conversion of the submerged shelf to bar and the exposed shelf to bench with plants like *Saccharum spontaneum*, *Vetiver zizanioides* and* Ipomoea carnea* have developed stable vegetated landforms along the study reaches (Fig. 2).

## Data and methodology

### GEE cloud computing and instream vegetation area assessment

**Figure 3:**
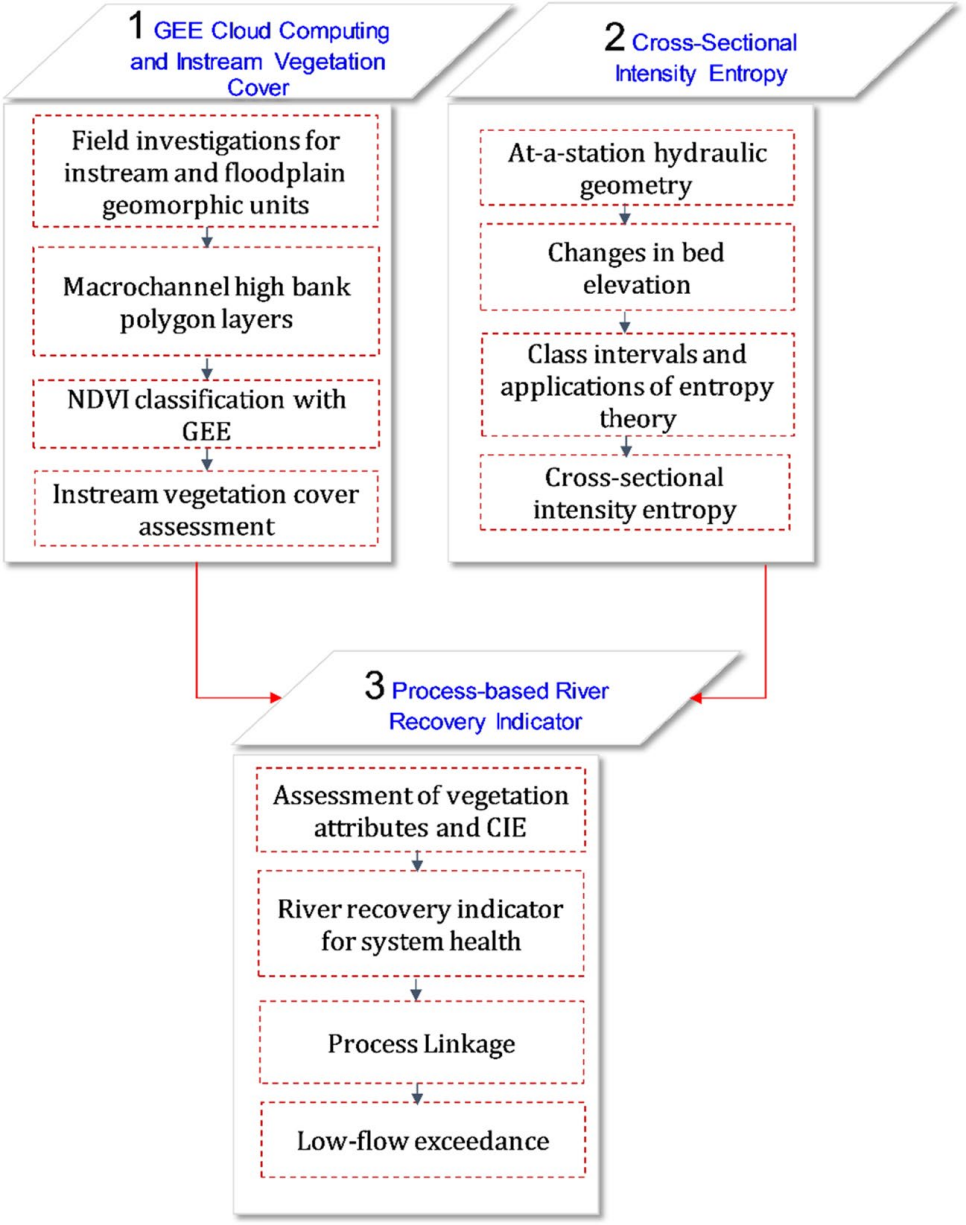
The methodological flowchart for evaluation of process-based recovery indicator.

The instream vegetation cover atop the bare bar and bench surfaces has been assessed with GEE for the last 35 years (1985–2020). Moreover, field investigations are carried out to identify the instream and floodplain geomorphic units along the study reaches. Then, the macrochannel bank lines are digitized based on field-observed floodplain signatures, and subsequent polygon layers are generated. The space between the macrochannel bank lines is selected as the fluvial corridor for instream vegetation cover assessment. The post-monsoon time refers to the low-flow hydrological condition during October–March. In this period, the flow is majorly concentrated in the thalweg (the deepest portion of the river) and therefore, geomorphic units like bar, bench, and instream vegetation cover are effectively captured from the field investigations and satellite imagery. The atmospherically corrected and orthorectified surface reflectance from Landsat 5 ETM, and 7 ETM + sensors are accessible by the GEE. For the given study period, these datasets are used to derive seasonal NDVI time series inside the fluvial corridor. Reflectance images in red and NIR bands of the electromagnetic spectrum from corresponding days of the acquisition are used to compute NDVI. Later, the temporal median of NDVI images is evaluated to derive a single seasonal NDVI representing the most general low-flow hydrological condition in the post-monsoon season. These computations are performed in the cloud platform of GEE, and final NDVI image time-series are exported for instream vegetation cover assessment (Fig. 3).

### Cross-sectional intensity entropy

Shannon’s information entropy theory is applied in this study, which is a measure of uncertainty or variability or disorder associated with the random variable $\mathrm{X}=\mathrm{X }({\mathrm{x}}_{1}, {\mathrm{x}}_{1}, {\mathrm{x}}_{1}\cdots \cdots \cdots ,{\mathrm{x}}_{\mathrm{n}})$^26^. Shannon^87^ defined the uncertainty of occurrence of an event x_i_ from the possible events in X as entropy H(X), which is given by

1 $$\mathrm{H}(\mathrm{x})= -\sum_{\mathrm{i}=1}^{\mathrm{n}}\mathrm{p}({\mathrm{x}}_{\mathrm{i}}){\mathrm{log}}_{2}(\mathrm{p}({\mathrm{x}}_{\mathrm{i}})).$$

here, p(x_i_) is the probability of x_i_, which is based on the empirical frequency of X values. The present study uses the concept of intensity entropy (IE) to investigate the seasonal (post-monsoon) variability associated with the macrochannel cross-sections. The cross-sectional intensity entropy (CIE) can be assessed as follows.

1. At-a-station hydraulic geometry dataset is obtained from India-WRIS, which consists of multi-decadal records (1989–2011). This dataset is collected with 10 m bin size intervals to understand the geomorphic aggradation (or degradation) associated with the instream geomorphic units.
2. Then, the alteration in channel bed elevation is computed from the reference year (1988), and the macrochannel geometry is further segregated into different zones based on the maximum breaks in slope.
3. The class intervals are carefully selected by examining the hydraulic geometries in post-monsoon seasons and decided based on the upper and lower bounds of the alterations in bed elevation. Therefore, all the possible variations in at-a-station hydraulic geometry and gradual macrochannel adjustments like thalweg shifting, chute development and the conversion of bar to bench are integrated to the analysis. The cross-sectional intensity entropy (CIE) is calculated as
  2 $$\mathrm{CIE }=-\sum_{\mathrm{i}=1}^{\mathrm{n}}\left(\frac{{\mathrm{f}}_{\mathrm{i}}}{\mathrm{N}}\right){\mathrm{log}}_{2}\left(\frac{{\mathrm{f}}_{\mathrm{i}}}{\mathrm{N}}\right)$$

where f_i_ is the number of elevation change values in a particular class (n) of a season to the total number of values in that season (N). The relative frequency is calculated as f_i_/N = p(x_i_). In this study, CIE is a measure of disorderness in the hydraulic geometry and distribution of available fluvial energy in the macrochannel system. CIE value reaches the maximum when the bed elevation changes are equally distributed between all classes and is minimum (= 0) only if all values fall into a particular class. Further, CIE symbolises the aggradation and degradation of instream geomorphic units at certain intervals and provides an understanding of the assemblage of landforms expected of river type and their past-to-present status and future trajectories. Fryirs et al.^15^ suggested various geomorphic adjustments like wide symmetrical channel to macrochannel, bank erosion to bench formation, actively widening to contracted channel, high width-depth ratio braid like low flow channel to well-defined low width-depth ratio thalweg as indicators between the pre-recovery and recovery states.

### Normalized river recovery index

Recovery is related to the improvement in geomorphic conditions, normally over the decadal period. It is also associated with the adjustments of the river to the prevailing boundary conditions like flow-sediment alteration, modifications in land-use practices, changes to instream fluvial cover etc. In addition, recovery is associated with the inherent sensitivity of the river, where each reach can be placed in its catchment context. The presence, absence or reconstruction of the assemblage of geomorphic units facilitate key indicators regarding the deterioration and recovery trajectory responses. Further, it is noted that the rate at which geomorphic units emerge provides a sign of the effectiveness of the recovery process. The present study has considered two important attributes of macrochannel rivers, i.e. vegetative measures and geomorphic disorderness, to provide a snapshot of how the system is performing at present. The normalized river recovery index (NRRI) as a system-state response is formulated as

3 $$\mathrm{NRRI }= \frac{{\mathrm{NORM}}_{\mathrm{VI}}-{\mathrm{NORM}}_{\mathrm{CIE}}}{{\mathrm{NORM}}_{\mathrm{VI}}+{\mathrm{NORM}}_{\mathrm{CIE}}}$$

where VI is vegetation intensity, CIE is cross-sectional intensity entropy, and NORM stands for normalization with maximum and minimum values. The vegetative measures often develop some fluvial landforms that trigger and engineer the river recovery process. In the present study area, instream vegetation growth is closely linked with CIE, where, after the stability of fluvial landforms, seed germination and gradual colonization have initiated. According to Thompson et al.^45^, with-in channel benches becomes more resilient after the vegetation growth and may reduce the disorderness in hydraulic geometry. Moreover, instream vegetation has improved the channel boundary resistance in present macrochannel settings, and the multiple inundation surfaces (bar, bench and macrochannel bank) have approached towards recovery with increased VI and gradual reduction of CIE.

### Process linkage

The recovery trajectory is enhanced or constrained by limiting factors and pressures. These limiting factors operate internally in the system and generally incorporate the changes to sediment supply, flow regime and instream vegetation cover. The pressures are the external agents and are integrated with environmental management policies and climate change etc. In the present study reach, it is well-established that effective discharge shapes the inset channel to carry a maximum portion of sediment load in the long term^26^. Further, the excess energy above the effective sediment transport level re-organises the chutes and diagonal bars close to the inset channel. Hence, a new process indicator—Low Flow Exceedance (LFE) is proposed to understand the impacts on the hierarchy of energy dissipation and river recovery processes in the morphological continuum zone of the macrochannels Ong and Tel. LFE signifies the number (N) of moderate-to-high flows exceeding the inset channel and submerging the instream vegetation zones at different platform levels. The LFE is proposed as

4 $$\mathrm{LFE}=\mathrm{ N}(\mathrm{Q}\ge {\mathrm{Q}}_{\mathrm{L}})$$

where N is number of days, Q is flow (in m^3^/s) and Q_L_ is carrying capacity of the inset channel (in m^3^/s).

## Results and discussion

### Longitudinal variation of instream vegetation cover

**Figure 4:**
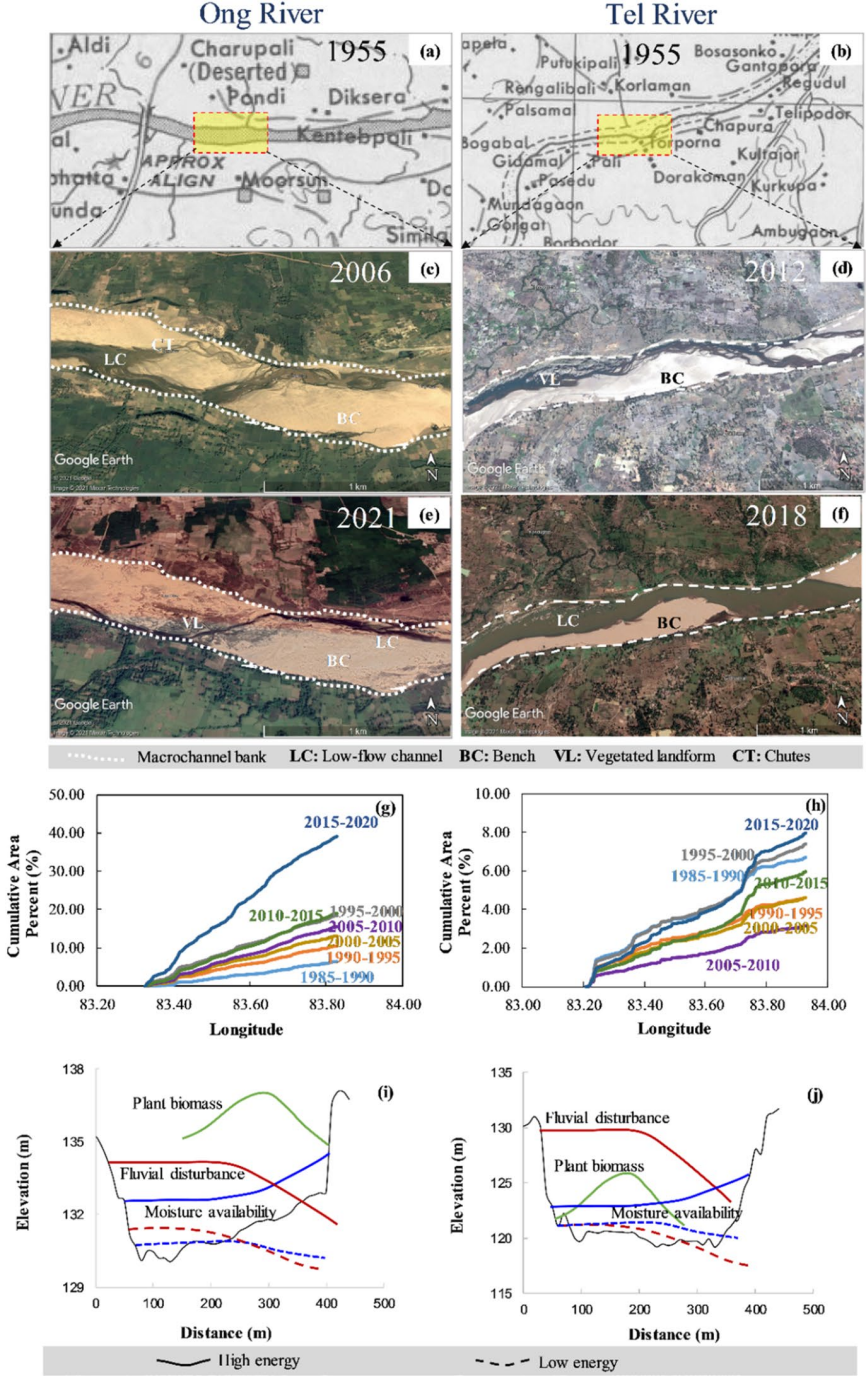
The Survey of India (SOI) toposheets for (**a**) the Ong (**b**) the Tel Rivers (Courtesy of the University of Texas Libraries, The University of Texas at Austin). The instream geomorphic units present in (**c**,**e**) the Ong and (**d**,**f**) the Tel Rivers. The longitudinal variation of instream vegetated landforms for last 35 years in (**g**) the Ong and (**h**) the Tel Rivers. The conceptual plots showing cross-sectional variability of plant biomass, fluvial disturbance and moisture availability in (**i**) the Ong and (**j**) the Tel Rivers.

No extensive changes to macrochannel position have occurred through bank erosion in the Ong and the Tel Rivers. The macrochannel bank is highly stable by riparian vegetation and the internal resistance of substrate lithology (Fig. 4a,b). The major changes are in terms of within-macrochannel adjustments like chute and floodways formations, bar and bench establishments and the gradual emergence of vegetated landforms. Figure 4c–h show the temporal changes in instream vegetation area alongside dominant geomorphic units present in the Ong and the Tel Rivers. In 1985–1990, both river systems have similar instream vegetation coverage, close to 7% of the total fluvial area. During this time slice, three to four selected areas are controlled by vegetated landforms close to the low-flow channels. The next time slice (1990–1995) marks contrasted changes in vegetation area, where the Ong shows an increased vegetation coverage up to 10.4% and the Tel exhibits slightly decreased area up to 4.7%. However, a steady growth in vegetation cover is observed in the subsequent time slice (1995–2000), and the Ong and the Tel have an instream vegetated areas of 18.4% and 7.39%, respectively. Between 1995 and 2000, the complete reach of the Ong river is affected by instream vegetation growth atop bar and bench surfaces, making it challenging to identify chute margins in some areas. The next decade is punctuated by major floods in 2001, 2003 and 2005, and a slight reduction in vegetation coverage was detected along the rivers. The decadal average vegetation cover is close to 14% and 4% along the Ong and the Tel, respectively. It should be noted that no wholesale change to vegetated landforms has occurred in the Tel river and periodic destruction, survival, and succession continued in the 2010s. In contrast, the Ong has experienced a drastic change in instream vegetation cover in the 2015–2020 time slice and increased the area from 19% to more than 30%.

The instream vegetation type, structure, density and position affect the process, form and dynamics of alluvial rivers at micro and macro scales^88–91^. In particular, the macrochannel river settings display multiple surfaces of varying inundation frequency and sediment trapping potential^39^. In the Ong, major vegetated landform zones and biomass density are observed on the bench surface and close to the low-flow channel (Fig. 4i). Furthermore, similar to the findings of Gurnell et al.^92^, the Ong river has experienced aggradation of the submerged shelf to lateral bars and the exposed shelf to a bench. These linear benches are natural repositories of seed germination and allow vegetation like *S. spontaneum*, *V. zizanioides* and *I. carnea* to colonize in the post-monsoon season.

A schematic representation of the distribution of soil moisture and fluvial disturbance suggests that vegetation patches have the support of perennially flowing water even at the lowest discharges. Therefore, the Ong River is affected by the development of vegetated patches close to the thalweg. Further, the periodic bio-geomorphological interactions have helped these vegetation patches to retain the upstream sediment, form deeper and narrower low-flow channels and eventually develop self-organized landforms. In contrast, the Tel resembles a confined channel with stable macrochannel banks, where high fluvial disturbances dominate the bio-geomorphological interactions (Fig. 4j). In this process, small-rooted plants have grown until they are destroyed by excessive fluvial erosion, leaving the small patches of deeper rooted plants to colonize the bare sediment of linear benches.

### Hydrological data analysis and variability in CIE and NRRI

**Figure 5:**
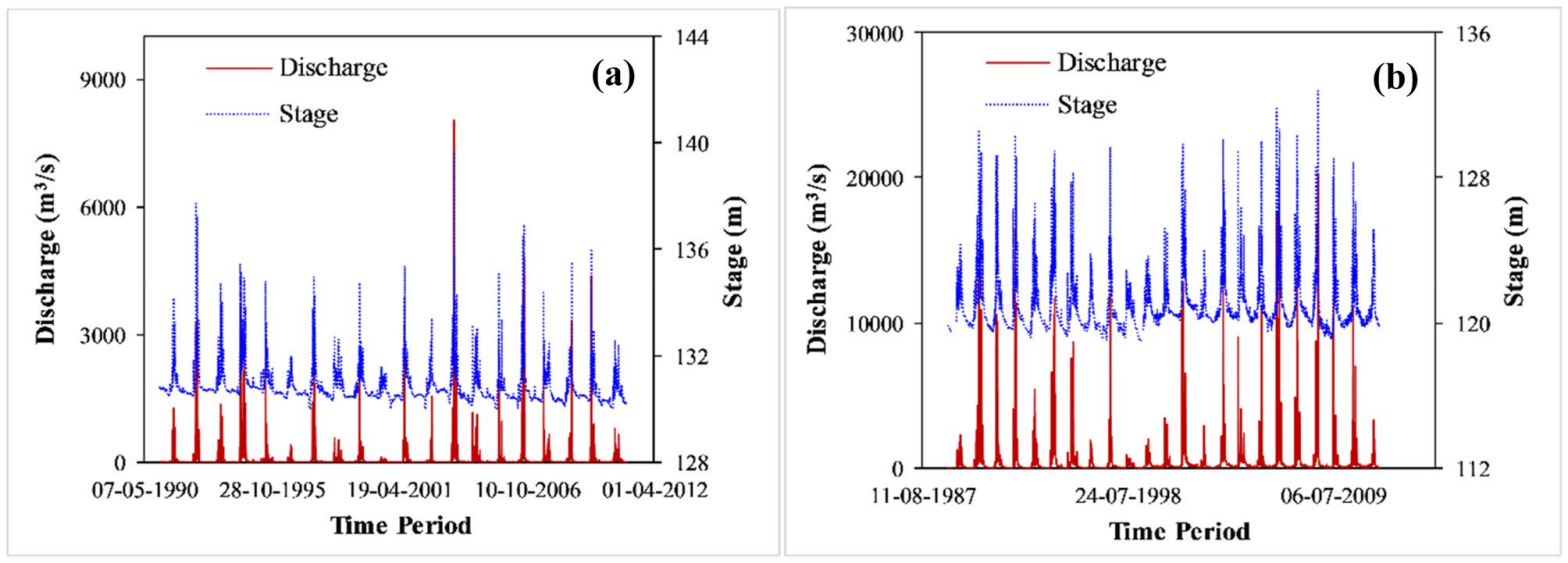
Variation of flow (in m^3^/s) and stage (in m- from MSL) along (**a**) the Ong (1991–2011) and (**b**) the Tel Rivers (1989–2011).

The oscillation of the south-west monsoon rainfall regime generates unimodal wet (June–September) and dry (October–May) seasons along the Ong and Tel Rivers (Fig. 5). The wet flows (floods and extreme events) are periodic, and a significant variation in discharge and stage (with respect to mean sea level-MSL) are observed along the study reaches. The transition between wet and low flows is characterized by moderate flows that provide a significant contribution to the bio-morphological activities. The time-averaged flow and stage along the Ong River are close to 130.80 m and 60.52 m^3^/s, respectively. However, the maximum water level and discharge are sufficiently large, and close to 139.53 m and 7916 m^3^/s, respectively (Fig. 5a). The Tel River observes a notable hydrological variability with time-averaged and maximum stages as 119.02 m and 132.7 m, respectively. The maximum observed discharge is 20,000 m^3^/s, which is considerably greater than a time-averaged value of 384 m^3^/s. Such extreme flow and water level variability highlight the ample in-channel space of macrochannel configuration, which can capture both high floods and extreme events (Fig. 5b).

**Figure 6:**
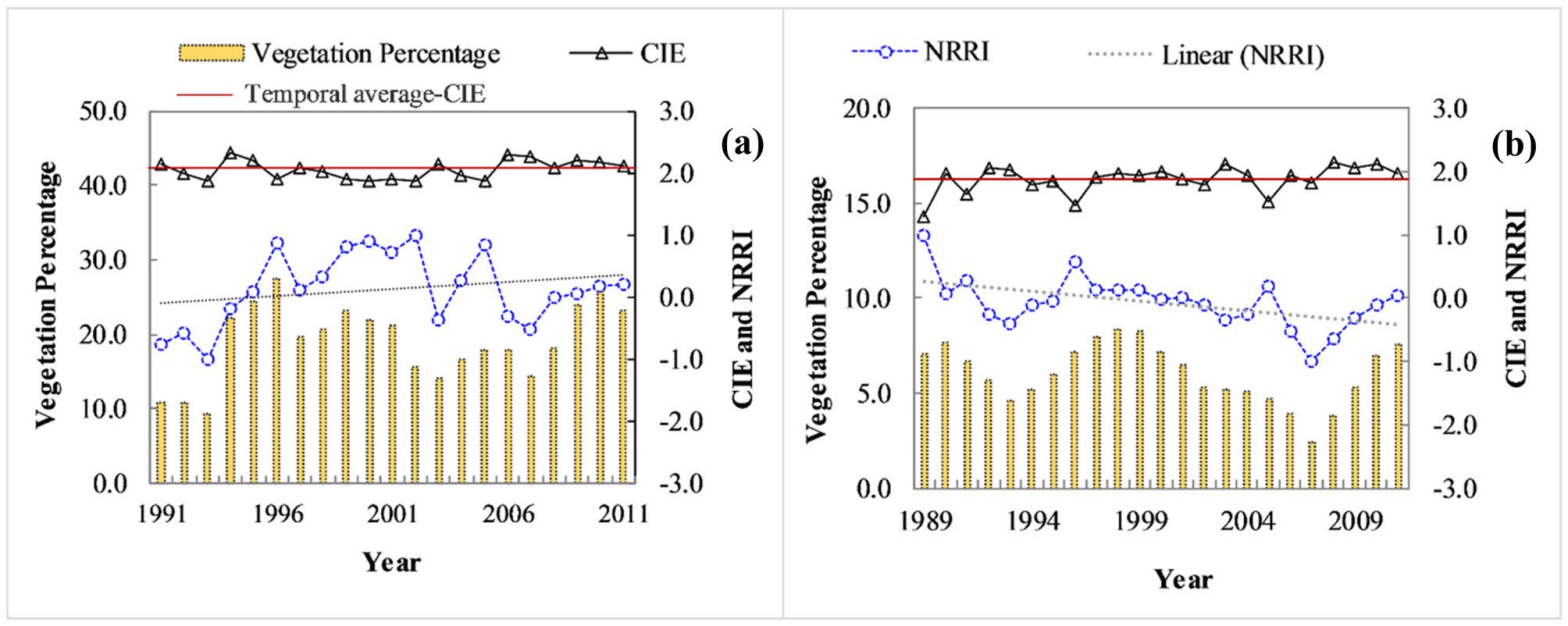
The temporal variation of yearly vegetation percentage, cross-sectional intensity entropy (CIE) and normalised river recovery index (NRRI) in (**a**) the Ong (**b**) the Tel Rivers. The linear trends of NRRI and temporal average-CIE are also shown.

Figure 6 shows the temporal variability of CIE along the Ong and Tel Rivers. It is observed that both river systems demonstrate continued hydraulic geometry adjustments via thalweg shifting, bar formation-sculpting-erosion and bench growth-destruction. In the Ong River, the temporal average of CIE is close to 2.05, whereas the Tel river has a slightly decreased CIE of 1.88. The maximum CIE in the Ong and Tel are close to 2.34 and 2.15, respectively. The Ong has a distinct minima of CIE variability in the early 2000s. Moreover, the Ong has displayed a continuous decreasing trend of CIE between 1997 and 2002 and after the extreme floods of 2003 and 2004, an increased CIE is noted in the late 2000s. In contrast, the Tel has no significant trend in CIE variability, and periodic fluctuations are noted over the entire study period.

The alteration in instream vegetation coverage and CIE have resulted in variable responses along the river recovery trajectory. In the regulated Ong, the temporal average of NRRI is close to 0.13, and it shows an inclining trend in the initial study period (1991–1992) due to the combined effect of decreased bar disorderness and slight reduction of instream vegetation cover on the bench surface (Fig. 6a). After the 1994 catastrophic flood, the morphological responses (in terms of CIE) has continued to decrease for the next 10 years. At the same time, the instream vegetation cover has increased from 10.76% (in 1992) to 21.06% (in 2001), and therefore, NRRI has increased from − 0.56 to 0.70. In contrast to the 1994-peak flood’s morphological and vegetation cover responses, the Ong has reacted in a different fashion to the 2003 and 2004 peak floods. A decrease in vegetation cover from 21.06 to 14.46% along with an increment in CIE from 1.91 to 2.27 has altered the NRRI from 0.7 to − 0.51. From this period onwards (2008–2011), the Ong river has accelerated the instream vegetation growth from 14.46 to 23.14% and gradually transformed into a macrochannel dominated by well-vegetated geomorphic units. In addition, CIE has followed a declining trend due to the occurrence of bench and transition to a depositional form of adjustments. The temporal average of NRRI in the Tel is close to − 0.06. The overall CIE trajectory establishes an initial increment of 51%, followed by a declining trend up to 1996, and finally, periodic fluctuations in the remaining years of the study period (Fig. 6b). The instream vegetation cover shows sporadic patterns of emergence, succession and decay and establishes an aerial coverage between 5 to 10%. Therefore, NRRI has observed a noticeable reduction (1.0 to − 0.1) between 1989 and 1994. After the 1994 extreme flood, diagonal bars have gradually transformed into vegetated landforms, and uniform aggradation of channel bed has improved the geomorphic condition, and therefore, NRRI was increased from − 0.24 to 0.01. The last time slice (2003–2011) is punctuated by two major floods in 2003 and 2004, and erosional form of adjustments like chute formation, channel straightening, sculpting of diagonal bars, and degradation of bench have continued for the next 3–4 years. In addition, the removal of smaller vegetated landforms and further succession in the subsequent years have helped the river to improve its geomorphic health and thus, NRRI has increased from − 1.0 to 0.05. This analysis establishes that the Tel river (unlike the Ong) in the Mahanadi catchment has not undergone the wholesale river change but has been adjusting within its behavioural regime.

The synthesis of literature pertinent to the form, process and evolution of macrochannel systems suggests that the channel-in-channel physiography provides sufficient space for both erosional activities (removal of a geomorphic unit, channel widening, bank mass failure, bend extension, the occurrence of chute channels, scour, and incision) and depositional processes (formation of new geomorphic units and accretion of sediment on multiple platform levels)^24,26,27,29,31,39,45,93^. The present study reaches along the Ong and the Tel are predominantly macrochannel, where geomorphic activities are limited to re-organisation of materials at different inundation surfaces. As per Phillips and Dyke^64^, a system state consists of a morphological structure held together by inter-woven form-process relationships. Therefore, in this study, CIE as a system state is concentrated on addressing the geomorphic adjustments at sub-bankfull stages. In the Ong, the linear bench gets partially eroded, washed away or accreted by floods which are discrete geomorphic disturbing events. The seldom realigning thalweg and bench evolution by sediment deposition and vegetation colonisation altered the system state of the Ong River. Furthermore, the post-disturbance states show a vegetated configuration that did not exist earlier. Thus, the gradual emergence of vegetated landforms and the declining trend of CIE can be referred to as a ‘state space expansion event’, coined by Phillips and Dyke^64^. However, in the Tel River, the fixed controls (macrochannel resistant banks) dominate over flux disturbances (floods) and result in a resilience system. The system state (CIE) and vegetation density oscillate about a mean structure, where the post-disturbance system state is either close to the pre-disturbance configuration or undergoes some minor adjustments.

In many instances, the vegetation succession and macrochannel evolution adhere to a linear, sequential logic^94^. Binary models have also gathered recent attention, which suggest oscillation of systems between two stable states^95,96^. The present study suggests that CIE and NRRI have followed fixed sequences of developmental stages in the study reaches unless extreme events are absent as disturbing agents. After the floods, the channel evolution is non-linear and follows complex relaxation paths. The catastrophic floods have accelerated the planform adjustments signaling a slow relaxation, less resilient and threshold-modulated system where CIE and riverine health is governed by extreme events in the Ong. In contrast, the Tel River has probably fast relaxation time and a filter-dominated system, where recurring impacts of catastrophic floods are quickly absorbed, and finally, a dynamically stable system is developed.

### River recovery and process linkage

**Figure 7:**
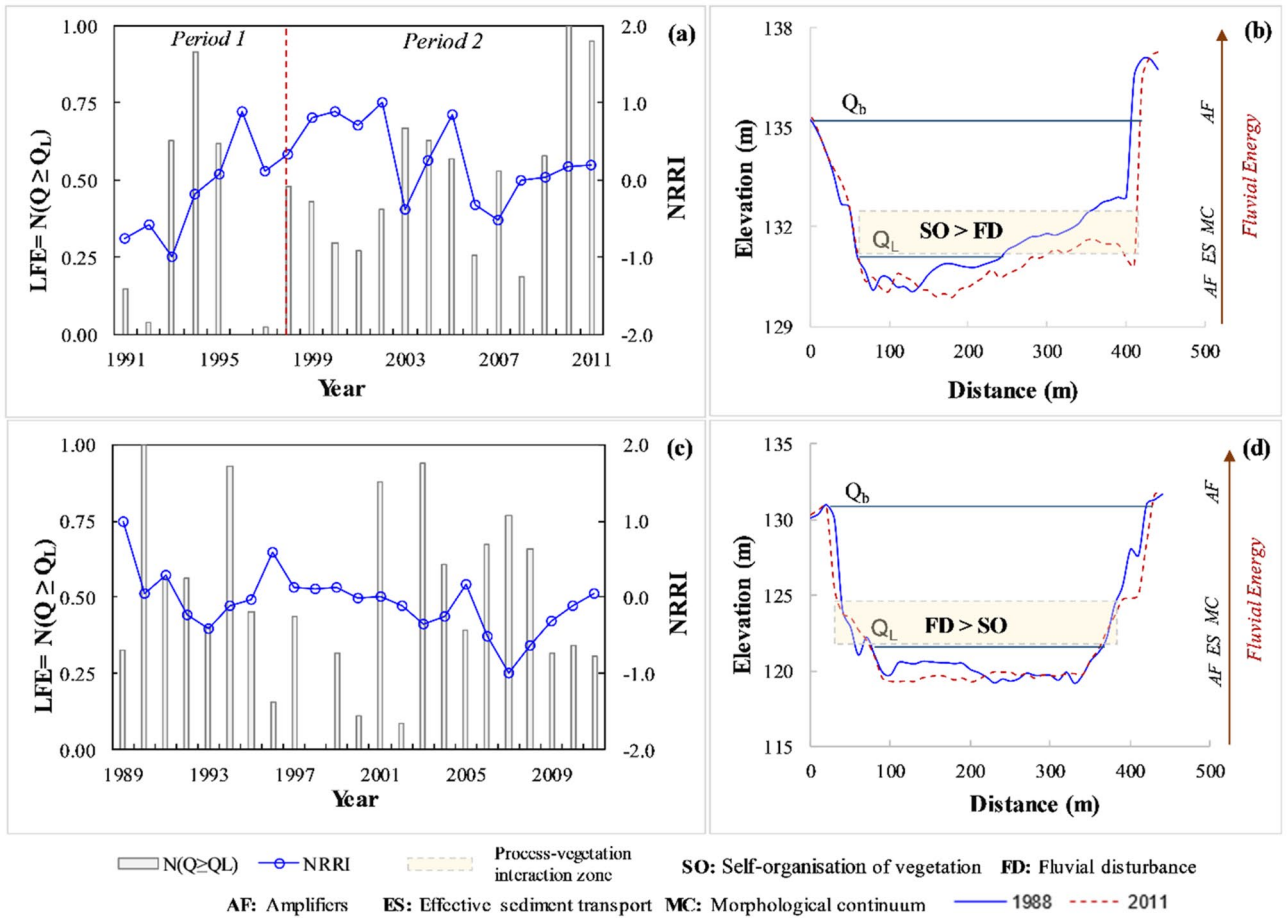
Relation between normalised low-flow exceedance (LFE = N(Q ≥ Q_L_)) and normalized river recovery index (NRRI) along (**a**) the Ong and (**c**) the Tel Rivers. The conceptual diagrams for the hierarchy of energy dissipation in macrochannel systems and dominant variables for the hotspot-zone of ecosystem engineering are also presented along (**b**) the Ong and (**d**) the Tel Rivers.

Figure 7 shows the relationship between process (N(Q ≥ Q_L_)) and recovery state (NRRI) along the study reaches. In the Ong, N(Q ≥ Q_L_) varies between 0 (Q ≥ Q_L_ ~ 40) to 1 (Q ≥ Q_L_ ~ 130) for the daily discharge hydrographs. The temporal variability of LFE indicates that the 1990s is punctuated by a distinct major flood in 1994, which has increased the LFE up to 0.91. The next time slice is characterized by fewer floods in 1996–1998 and increased moderate floods in 1999–2002. The 2000s have witnessed frequent floods exceeding the low-flow channel and instigating major morphological adjustments. At the end of the study period (2007–2011), LFE has an accelerated increase from 0.53 to 0.95, suggesting alteration to both flow-regime and low-flow channel morphology in the Ong river. For the Tel River, N(Q ≥ Q_L_) fluctuates between 0 (Q ≥ Q_L_ ~ 9) to 1 (Q ≥ Q_L_ ~ 91) for the daily discharge hydrographs. Figure 7b displays three-time slices divided based on LFE variability, where the first period (1989–1994) is defined by frequent floods overtopping the low-flow channel. However, the second (1995–2000) time slice is characterised by the small number of floods exceeding the thalweg (average LFE of 0.25), and in the last period (2001–2011), the average LFE had increased up to 0.54, signalling a relative change in the flow regime.

As suggested by Pradhan et al.^26^, most of the geomorphic adjustments occur below the macrochannel bankfull level, and the river goes through a transition to accommodate the effective discharge in the inset channel. In addition, excess energy above the effective sediment transport re-organises chutes and diagonal bars close to the low-flow channel. The present research finding is pertinent to rivers with transitional channel patterns, where the river fluctuates the planform state between sinuous and weakly braided. This study highlights the hierarchy associated with the (re)organisation process in the macrochannel river system and its contribution to the river recovery state (Fig. 7b,d). For example, the inter-comparison of LFE and NRRI trends showed that two distinct time periods are observed on the basis of low-flow exceedance number variability in the Ong River (Fig. 7a). In period 1 (1991–1998), LFE and NRRI are less related to each other and in period 2 (1999–2011), both parameters are well-correlated. In period 1, the Ong has high fluctuations in small-moderate flood magnitudes, and CIE has initial variability due to the bar disorderness, followed by an initiation of the decreasing trend. This morphological activity is combined with the erratic instream vegetation growth pattern and has generated a ‘lagged system’, where LFE and NRRI are not correlated. However, in period 2, both parameters have followed a similar trend attributed to the gradual alteration in CIE and subsequent stabilisation of vegetated landforms. Hence, the macrochannel system may have switched to an ‘event-driven’ state, where floods exceeding the low-flow channel possess a direct impact on the river recovery trajectory. In the Tel river, the vegetation cover is significantly low (5–10%), and this large macrochannel system has witnessed extreme flow variability (20–20,000 m^3^/s) in the 1990s. Such drastic changes to the flow regime have triggered morphological adjustments and have kept the channel perpetually in transition between sinuous and weakly braided states. Therefore, for such large channel-in-channel physiography, the relationship between LFE and NRRI is challenging to establish and may entail the integration of additional process variables.

The geomorphic threshold is one of the fundamental concepts related to the existence of discrete planform states (straight-meandering-braided)^97–100^. However, the presence of intermediate, transitional channel patterns within the behavioural regime suggests the presence of a continuum in the macrochannel systems^26^. Fluvial controls like flow strength index, bank erodibility and sediment supply are related to the morphological continuum^101,102^. In our river systems, amplifiers (AS) (both low-flow and extreme events) and effective sediment transport (ES) constitute the hierarchy of energy dissipation. Furthermore, the zone of the morphological continuum lies just above the effective sediment transport stage level (Fig. 7b,d), which incorporates the major form of geomorphological adjustments. As per Gurnell et al.^103^, the ‘critical zone’ for physical ecosystem engineering exists within the area of the river corridor that is perennially inundated. The adjacent areas are subjected to frequent inundation and significant shear stresses and erosion–deposition of sediment. Therefore, in the present study, the zone of morphological continuum and critical areas of plant-hydrogeomorphology interactions overlap within the sub-macrochannel bankfull level (Fig. 7). This bio-geomorphological interaction can also affect the changes in river planform type and future directions^104,105^. Our findings suggest that the instream vegetation growth close to the inset channel affects (or controls) the morphological activities in terms of cross-sectional disorderness. Hence, within the continuum zone, the vegetation patches can grow, self–organise (SO) and expand to form enlarged vegetated landforms or become smaller and widely spaced under the influence of fluvial disturbances (FD). If SO starts to dominate over FD, the vegetation growth reduces the CIE variability, stabilizes the low-flow channels and increases NRRI (Fig. 7b). In contrast, if chutes and bar disorderness dictate SO through frequent fluvial disturbances, the rate of planform fluctuations increases between the end-points of the morphological continuum (sinuous and weakly braided states) (Fig. 7d). The channel evolution models are also associated with channel adjustments along the four degrees of freedom (cross-section, planform, bed and slope)^106^. The present paper emphasizes the presence of instream vegetation as an additional degree of freedom, which further controls the hierarchy of energy dissipation and morphological continuum in the macrochannel settings.

In the era of big data and cloud computing, assessment of system state indicator-CIE and process-based indicator-NRRI will certainly help to comprehend the fluvial trajectory and develop recovery enhancement approaches. Further, this framework will be helpful for planning and prioritizing recovery indicators which can reduce the cross-sectional disorderness and promote improvements in river health. However, many fluvial systems in developing countries are poorly gauged, and assessing CIE and system disorderness with the continuous hydraulic and hydrological dataset is challenging. Therefore, the delicate balance between big data and remote sensing-based tools with archival benchmark information, ‘place-based understanding’ and ‘reading the landscape’ frameworks (Brierley et al. 2013)^107^ will be key to capture and inform process-form relationships of fine-scale geomorphic units.

## Conclusion

The present study has developed a process-based river recovery indicator for anthropogenically disturbed macrochannel river systems. The major conclusions of this study are as follows:

- The disorderness in bed-elevation at sub-bankfull stages is effectively captured by a system state indicator-CIE. The temporal variation of CIE has further addressed the existence of both threshold-modulated and filter-dominated systems in macrochannel settings.
- Vegetation density and CIE integrated recovery indicator (NRRI) symbolize river health for channel-in-channel fluvial systems. The present study suggests that CIE and NRRI have followed fixed linear sequences of developmental stages unless extreme events are absent as disturbing agents.
- A gradual decline in CIE and subsequent stabilization of vegetated landforms can develop an ‘event-driven’ state, where floods exceeding the low-flow channel (LFE) possess a direct impact on the river recovery trajectory.
- Finally, the dominance between self-organization of vegetated landforms and fluvial disturbances develops an additional degree of freedom and further decides the recovery state of macrochannel and planform fluctuations between the end-points of the morphological continuum (sinuous and weakly braided states).

Natural rivers are gradually subjected to altered flow-sediment conditions through various natural and anthropogenic stressors. Therefore, predicting the trajectory of recovery potential is crucial in river restoration programs. The present study presented a novel approach to predict the system state and recovery potential in anthropogenically disturbed macrochannels. This work can be further extended to rivers with different morphological settings and channel pattern types to better understand the fluvial dynamics.

## Data Availability

The dataset used in this study are openly available at- Landsat Imagery (https://developers.google.com/earth-engine/datasets), cross-sectional geometry and hydrological data (https://indiawris.gov.in/wris/), and survey of India Toposheet (https://maps.lib.utexas.edu/maps/ams/india/).
